# RNAi therapy targeting KRAS in combination with chemotherapy for locally advanced pancreatic cancer patients

**DOI:** 10.18632/oncotarget.4183

**Published:** 2015-05-19

**Authors:** Talia Golan, Elina Zorde Khvalevsky, Ayala Hubert, Rachel Malka Gabai, Naama Hen, Amiel Segal, Abraham Domb, Gil Harari, Eliel Ben David, Stephen Raskin, Yuri Goldes, Eran Goldin, Rami Eliakim, Maor Lahav, Yael Kopleman, Alain Dancour, Amotz Shemi, Eithan Galun

**Affiliations:** ^1^ The Sackler School of Medicine, The Chaim Sheba Medical Center, Tel Aviv University, Tel Aviv, Israel; ^2^ Silenseed Ltd., Jerusalem, Israel; ^3^ Sharett Institute of Oncology, Hadassah-Hebrew University Medical Center, Jerusalem, Israel; ^4^ The Gastroenterology Institute, Shaare Zedek Medical Centre, Jerusalem, Israel; ^5^ Institute of Drug Research, School of Pharmacy-Faculty of Medicine, Center for Nanoscience and Nanotechnology and The Alex Grass Centre for Drug Design and Synthesis, The Hebrew University of Jerusalem, Jerusalem, Israel; ^6^ MediStat Ltd., Jerusalem, Israel; ^7^ Hadassah-Hebrew University Medical Centre, Jerusalem, Israel; ^8^ The Gastroenterology Institute, Shaare Zedek Medical Center, Jerusalem, Israel; ^9^ Gastroenterology Institute, Hadassah Hebrew University Medical Center, Jerusalem, Israel; ^10^ The Goldyne Savad Institute for Gene Therapy, Hadassah Hebrew University Medical Center, Jerusalem, Israel

**Keywords:** Pancreatic cancer, KRAS, overall survival, siRNA, polymer implant

## Abstract

**Purpose:**

The miniature biodegradable implant *siG12D-LODER™* was inserted into a tumor and released a siRNA drug against KRAS(G12D) along four months. This novel siRNA based drug was studied, in combination with chemotherapy, as targeted therapy for Locally Advanced Pancreatic Cancer (LAPC).

**Methods:**

An open-label Phase 1/2a study in the first-line setting of patients with non-operable LAPC was initiated. In this study patients were assigned to receive a single dose of *siG12D-LODERs*, in three escalating dose cohorts (0.025mg, 0.75mg and 3.0mg). Gemcitabine was given on a weekly basis, following the *siG12D-LODER^TM^* insertion, until disease progression. The recommended dose was further examined with modified FOLFIRINOX. The follow up period was eight weeks and survival until death.

**Results:**

Fifteen patients with LAPC were enrolled. Among the 15 treated patients, the most frequent adverse events observed were grade 1or 2 in severity (89%); five patients experienced serious adverse events (SAEs). In 12 patients analyzed by CT scans, none showed tumor progression, the majority (10/12) demonstrated stable disease and two showed partial response. Decrease in tumor marker CA19-9 was observed in 70% (7/10) of patients. Median overall survival was 15.12 months; 18 month survival was 38.5%.

**Conclusions:**

The combination of *siG12D-LODER™* and chemotherapy is well tolerated, safe and demonstrated a potential efficacy in patients with LAPC. NCT01188785

## INTRODUCTION

### Pancreatic cancer

Pancreatic ductal adenocarcinoma (PDAC) is the most common pancreatic neoplasm, responsible for 90% of pancreatic cancer cases [1]. Until recently Gemcitabine was considered the main treatment option. Recent progress in the treatment of metastatic PDAC has been shown with the addition of nab-paclitaxel to Gemcitabine [2] and the introduction of FOLFIRINOX (Leucovorin, Fluorouracil, Irinotecan and Oxaliplatin) [3]. FOLFIRINOX substantially improves overall survival (OS) compared to Gemcitabine, however, it has a high toxicity profile and hence only suitable for a subset of patients. About 80% to 85% of patients with pancreatic cancer have advanced disease at the time of diagnosis, i.e. stage III (LAPC) or stage IV (metastatic), and are not candidates for surgical curative intervention. Several autopsy series demonstrated that 30% of patients presenting with stage III disease died without evidence of distant metastases [4].

Treatment options for LAPC include several chemotherapy regimens (Gemcitabine, Gemcitabine and Erlotinib, Gemcitabine and Nab-Paclitaxel, or FOLFIRINOX) with or without radiation. It is still unclear whether chemoradiation for LAPC patients is warranted. Chauffert et al studied initial chemoradiotherapy (intermittent Cisplatin and infusional 5-FU) followed by Gemcitabine *vs*. Gemcitabine alone in patients with LAPC and observed a median survival of 8.6 months for the chemoradiation arm *vs*. 13 months for chemotherapy alone [5]. Loehrer et al. showed that treatment of patients with Gemcitabine alone or with radiation led to survival of 9.2 and 11.1 months respectively [6]. Herman et al. demonstrated that treatment of LAPC patients with chemoradiotherapy (50.4 Gy with concurrent fluorouracil), with or without TNFerade, led to median survival of 10.0 months in both treatment arms. Eighteen month survival was 13.3% (standard of care (SOC)) *vs*. 18.2% (SOC+TNFerade) [7]. Progression Free Survival (PFS) was 7.0 months (SOC) *vs*. 6.8 months (SOC+TNFerade) and 18 month PFS was 6.6% (SOC) *vs*. 8% (SOC+TNFerade) [7]. Blazer et al. reported that non-resectable FOLFIRINOX-treated patients showed median OS and PFS of 12.7 and 8 months respectively [8].

Epithelial carcinogenesis is typically associated with the accumulation of mutations and genetic lesions, leading to the activation of oncogenes and inactivation of tumor suppressor genes. Mutated KRAS was proposed to be a hallmark of pancreatic cancer [9] since this protein is mutated in more than 90% of PDACs [10]. Mounting literature data suggest that the KRAS mutations may be early events in the molecular pathological cascade leading to PDAC [11]. The vast majority of KRAS mutations in adenocarcinoma of the human pancreas are gain-of-function mutations, in which most of these occur in codon 12. Among the codon 12 mutations the most abundant is substitution of the Glycine for Aspartate (G12D) [9, 12, 13]. In PDAC cancer cells are addicted to expression of the mutated KRAS [9, 14]. Still, more than 30 years of research have not yielded a specific therapy directly targeting mutated KRAS. The challenges are attributed to the picomolar affinity of this oncogene for GTP/GDP [15, 16] and failure to target any relevant allosteric regulatory sites. Recently, the maturing field of mRNA targeting by RNA interference (RNAi) has proven to be a highly potent alternative compared to the more general protein inhibition approach. It was previously shown that suppression of KRAS expression by RNAi led to growth inhibition of PDAC cells *in vitro* and PDAC-originated tumors *in vivo* in mice [17, 18].

### RNAi-based anti-mutated KRAS treatment for patients with LAPC

To address the unmet need for an efficient non-toxic treatment for patients with LAPC, and to convert the potential of RNAi into an anti-cancer therapy, Silenseed Ltd. has developed the *LODER^TM^* (Local Drug EluteR). *LODER^TM^* presents a novel solution to the major challenges of oligonucleotide therapeutics and RNAi for a large number of diseases including solid tumors. Such challenges include delivery of RNAi-based drugs and achievement of prolonged activity at a tolerated dose at the target site. The *siG12D-LODER^TM^* is a miniature biodegradable polymeric matrix that encompasses anti-KRASG12D siRNA (siG12D). *siG12D-LODER^TM^* is designed to provide a slow and prolonged local drug release within the tumor over several months, while ensuring protection of the siRNA drug from degradation. It can be inserted and placed into pancreatic tumor using a standard endoscope ultrasound (EUS) biopsy procedure. Here, we present the use of *siG12D-LODER^TM^* as a new therapeutic modality targeting mutated KRAS in combination with the currently available treatments including Gemcitabine or FOLFIRINOX.

Our treatment of LAPC with *siG12D-LODER^TM^* is based on accumulated pre-clinical evidence [19]. The results of these studies proved that (a) *siG12D-LODER^TM^* efficiently overcomes current siRNA delivery obstacles related to systemic delivery; (b) the LODER's pharmacokinetics (PK) enable dose reduction by orders of magnitudes and eliminates toxicity; (c) the strategy of local KRAS targeting by *siG12D-LODER^TM^* can be effectively used to inhibit PDAC cell proliferation [19].

On the basis of these promising pre-clinical results, a first in-human Phase 1/2a clinical study of *siG12D-LODER^TM^* in combination with chemotherapy was initiated for patients with locally advanced PDAC. The primary objectives were to assess safety and tolerability as well as to define a recommended phase 2b dose (RP2D) of *siG12D-LODER^TM^*. Secondary objectives included measuring the efficacy potential of *siG12D-LODER^TM^* (though limited by a single dosing design), the antitumor effects on the basis of tumor response, CA19-9 levels, Time To Metastasis (TTM), PFS and OS.

## RESULTS

### LODER*^TM^* characteristics

*LODER^TM^* is a miniature biodegradable matrix designed by Silenseed, allowing slow and prolonged local release of the encapsulated drug. *LODER^TM^* was developed to be inserted and placed into solid tumors using a standard biopsy procedure. *siG12D-LODER^TM^* was designed to release anti-KRASG12D siRNA (siG12D) within the pancreatic tumor for four months. *siG12D-*LODER^TM^ was shown to efficiently protect the encapsulated siRNA against enzymatic degradation, while releasing the drug within a tumor *in vivo* in mice (reference 19 and Figure 2A). The selected *LODER^TM^* matrix components are all FDA Generally Recognized As Safe (GRAS) materials, and the active agent siG12D was found to be non-toxic in all doses *in vivo*. Serial histopathological examination demonstrated that the drug covers the entire tumor mass typically within a week (Figure 2). Our data are consistent with previous evidence of uptake of naked siRNA within solid tumors [20]. Direct effects of KRAS expression inhibition on the mRNA and protein levels, as well as indirect effects of inhibition of tumor growth and induction of tumor cell death, were detected in the entire tumor mass (reference 19 and Figure 2B). Tumor growth was slowed as demonstrated in diverse *in vivo* mice models (subcutaneous and orthotopic), following implantation of *siG12D-LODER^TM^* and the mice survival rates were significantly improved [19]. In *siG12D-LODER^TM^*-treated tumor tissue, apoptosis of tumor cells and tissue necrosis were found to be widespread after typically one week in the entire tumor (reference 19 and Figure 2B).

In addition to local effects on the tumor, we have shown in preclinical studies that siG12D slows tumor cell migration (Supplementary Figure 1C) and inhibits Epithelial to Mesenchymal Transition (EMT) (data not shown). Consistent with the above observations, we have found that mice treated with *siG12D-LODERs* did not develop metastases as compared with two thirds of untreated controls and *empty-LODER^TM^*-treated groups, which developed metastases (Supplementary Figure 1D).

The siG12D dosage in humans was selected based on the *in vivo* results in mice (Supplementary Figure 1A-1B). We have estimated a maximum dose of 3mg of siRNA above which RNAi saturation occurs.

*siG12D-LODER^TM^* also affects the second most abundant KRAS mutation, G12V, which implies that the *siG12D-LODER^TM^* efficacy is not restricted to the G12D mutation (data not shown). We further showed that siG12D provides additive benefit when used concomitantly with SOC chemotherapy, i.e. Gemcitabine or FOLFIRINOX (Supplementary Figure 2A-2B).

Systemically, the dose levels of the siG12D in the peripheral circulation are below detection levels, making measurements of (systemic) pharmacokinetics (PK) and pharmacodynamics (PD) impractical. However, the pharmacokinetics of siG12D drug in the tumor tissues can be assessed *in vivo*. We have studied PK and drug transport in a subcutaneous pancreatic tumor model. The amount of intercellular siG12D molecules that penetrate into cells was assessed by detection of antisense strand of the molecule in the treated tissue using absolute quantitative Real-Time PCR. siG12D molecules were detected at distances extended by typical velocity of 1mm/day until complete tumor coverage after a week. For example, in mouse subcutaneous model tumors of more than 1.7 cm in diameter were covered by siG12D molecule within a week (Figure 2A and data not shown). The PK results after a month are similar to the results after 7 days (data not shown), confirming a steady state of the drug distribution from *LODER^TM^*. These direct measurements of the drug transport to extended distances are in line with results of the drug effect on the entire tissue. For example, Figure 2B presents tumor tissue necrosis detected in the entire tumor area one week after *siG12D-LODER^TM^* implantation (in this example, the tumor is extended to about 1.7cm in diameter).

Shemi et al (in preparation, 2015) explores these results and their implications on drug transport in human solid tumors. They proposed a transport model which simulated drug delivery rates and demonstrated that drug delivery originating at the inner core of a tumor differs from systemic delivery. In systemic delivery drugs (or nanocarriers) need to cross the artery walls and penetrate into the inner tumor upstream, against the interstitial fluid pressure gradient. The transport of siRNA drug from the *LODER^TM^* surface into the entire tumor tissue was analysed and was explained by convection dominated transport (*Péclet* number > 1, where the *Péclet* number reflects the rate of convection of a flow to its rate of diffusion). The drug is distributed from the inner core outwards in the tortuous tissue with effective velocity of 1mm/day, and reaches the entire tumor mass in approximately one week.

**Figure 1:**
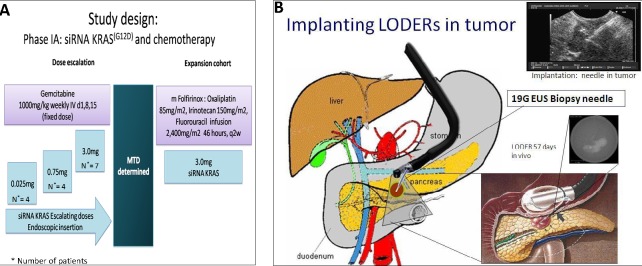
**A.** Study design. **B.** *siG12D-LODER^TM^* is placed with Endoscopic US biopsy needle.

**Figure 2:**
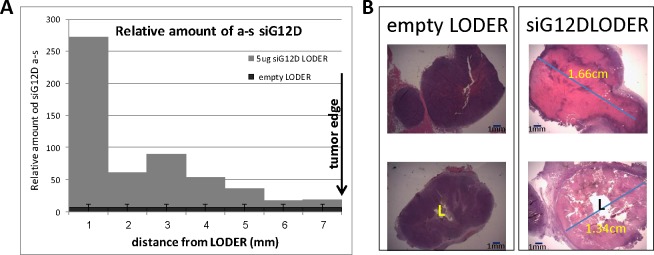
siG12D drug covers the entire tumor tissue within one week Subcutaneous tumors of pancreatic PancO2 origin were treated with empty-*LODER^TM^* or *LODER^TM^* containing 5μg siG12D. Seven days post-implantation mice were sacrificed, tumor tissue was formalin-fixed, paraffin embedded and cut to slices of 5μm. **A.** The graph depicts relative amounts of antisense siG12D strand, measured by Relative Quantitative Real-Time PCR, at certain distances from the *LODER^TM^* border. The results were normalized to RNU6 and calibrated to untreated control. **B.** Representative tumor tissue, H&E stained, seven days post implantation.

### Patient characteristics

The demographic and baseline characteristics of the study subjects in each treatment group are presented in Table 1. The median patient age was 70 years (range 52.1-85.3), where 30% of patients were above age of 74. Fifteen patients with locally advanced PDAC were enrolled across three dose levels: 0.025mg, 0.75mg and 3.0mg. Of these, four patients received a low dose 0.025mg of *siG12D-LODER^TM^*, four patients received 0.75mg (i.e. 0.375mg x 2 *siG12D-LODER^TM^*) and seven received 3mg (i.e. 0.375mg x 8 *siG12D-LODER^TM^*) (Figure 1A). Fourteen of the patients received concurrent SOC and one patient received no chemotherapy (Table 1). All 15 patients completed the study. Two patients were omitted from study but followed for safety due to metastatic disease detected on day one post *siG12D-LODER^TM^* implant imaging. The average age of the study subjects at screening was 68.8 ± 9.0 years (range: 52.1-85.3). Eight subjects were males (53.3%).

**Table 1:** Baseline patient cohort characteristics

	siG12D LODER^TM^ dose			
Parameter	0.025 mg N=4	0.75 mg N=4	3 mg N=7	All N=15
Mean Age ± SD*, years	65.8 ± 12.5	67.2 ± 6.0	71.5 ± 8.9	68.8 ± 9.0
**Median, years**	64.4	66.9	70.8	69.9
**(Range)**	(52.1–82.0)	(60.9–74.2)	(57.6–85.3)	(52.1–85.3)
**Gender:**				
** Male, N (%)**	3 (75.0)	1 (25.0)	4 (57.1)	8 (53.3)
** Female, N (%)**	1 (25.0)	3 (75.0)	3 (42.9)	7 (46.7)
**Smoker:**				
** Current** (%)	1 (25.0)	-	-	1 (6.7)
** Past** (%)	1 (25.0)	-	-	1 (6.7)
**Concomitant chemotherapy**				
**Treatment**	*siG12D LODER^TM^* dose			
	0.025 mg N=4	0.75 mg N=4	3 mg N=7	All N=15**
**Gemcitabine**	2	4	3	9
**Oxaliplatin + Irinotecan +Fluorouracil**	-	-	4	4
**Gemcitabine + Erlotinib + Oxaliplatin**	1	-	-	1
**None**	1	-	-	1

* SD=standard deviation

** Number of patients enrolled (Two patients were omitted due to metastatic disease detected on day 1 post *siG12D-LODER^TM^* implantation)

### Concomitant chemotherapy

Nine patients received concomitant Gemcitabine as SOC (50.0% of patients in the low treatment group, all patients in the mid-treatment group and 42.9% of patients in the high-treatment group). One patient received the Gemcitabine + Erlotinib + Oxaliplatin combination and one patient refused to receive any concomitant chemotherapy during the study. Four patients in the high-treatment (57.1%) group received modified FOLFIRINOX (Table 1).

### Safety

No DLTs (Dose Limiting Toxicity) events were observed during the study. No MTD (Maximal Tolerated Dose) was observed in the study. All 15 patients reported a total of 125 AEs. 111 of reported AEs (89%) were grade 1 and 2, which were transient and resolved. The most common AEs (grade 1-2) were diarrhea and abdominal pain which were reported by seven patients (46.7%), nausea and fatigue were reported by six (40%) and five (33.3%) patients respectively. 13 grade 3 and one grade 4 severe AEs were reported by 10 (66.7%) subjects. The most common grade 3-4 AEs were neutropenia and cholangitis reported by 3 patients and 2 patients respectively.

AEs were separately analyzed as “procedure-related” and “drug-related” (Table 2). Eight procedure-related AEs (Table 2) were reported by four patients (26.7%), of them, seven AEs were reported by three patients in the 3mg treatment group and one in the 0.025mg treatment group. Three procedure-related AEs in two patients were grade 1-2 (38%). Only one AE (grade 3 cholangitis) was considered as definitely related to the study procedure, as it was observed one day after procedure. To note, the single grade 3 AE related to pancreatitis, which was reported as ‘possibly related’ to the study procedure, seems to be unlikely related to procedure as it was identified 23 days after *siG12D-LODER^TM^* implantation. In general, our experience both in animals [19] and humans is that there are no signs of pancreatitis. Even in cases where patients had previous resection (partial pancreatectomy), there is very rarely pancreatitis.

**Table 2:** Adverse events summary of AEs occurring in ≥ 2 patients in any treatment arm by preferred term

		0.025 mg N=4		0.75 mg N=4	3 mg N=7		All N=15
	**N**	**(%)**	**N**	**(%)**		**N**	**(%)**
**Diarrhoea**	1	(25.0)	1	(25.0)		7	(46.7)
**Abdominal pain**	1	(25.0)	1	(25.0)		6	(40.0)
**Nausea**	1	(25.0)	1	(25.0)		6	(40.0)
**Fatigue**	-	-	1	(25.0)		5	(33.3)
**Vomiting**	-	-	2	(50.0)		4	(26.7)
**Pyrexia**	2	(50.0)	1	(25.0)		3	(20.0)
**Rash**	1	(25.0)	2	(50.0)		3	(20.0)
**Back pain**	1	(25.0)	-	-		3	(20.0)
**Anaemia**	-	-	2	(50.0)		2	(13.3)
**Neutropenia**	-	-	2	(50.0)		2	(13.3)
**Influenza like illness**	-	-	-	-		2	(13.3)
**Infusion related reaction**	-	-	-	-		2	(13.3)
**Platelet count decreased**	-	-	-	-		2	(13.3)
**Alopecia**	-	-	-	-		2	(13.3)

Summary of procedure-related AEs									
Preferred term	Relationship	0.025 mg N=4		0.75 mg N=4		3 mg N=7		All N=15	
		N	(%)	N	(%)	N	(%)	N	(%)
**Pancytopenia**	Possibly related	-	-	-	-	1	(14.3)	1	(6.7)
**Abdominal pain**	Possibly related	-	-	-	-	2	(28.6)	2	(13.3)
**Colonic obstruction**	Possibly related	-	-	-	-	1	(14.3)	1	(6.7)
**Constipation**	Possibly related	-	-	-	-	1	(14.3)	1	(6.7)
**Pancreatitis**	Possibly related	1	(25.0)	-	-	-	-	1	(6.7)
**Cholangitis**	Definitely related	-	-	-	-	1	(14.3)	1	(6.7)
**Renal failure**	Possibly related	-	-	-	-	1	(14.3)	1	(6.7)

Summary of drug-related AEs									
Preferred term	Relationship	0.025 mg N=4		0.75 mg N=4		3 mg N=7		All N=15	
		N	(%)	N	(%)			N	(%)
**Pancytopenia**	Possibly related	-	-	-	-	1	(14.3)	1	(6.7)
**Abdominal pain**	Possibly related	-	-	-	-	2	(28.6)	2	(13.3)
**Colonic obstruction**	Possibly related	-	-	-	-	1	(14.3)	1	(6.7)
**Constipation**	Possibly related	-	-	-	-	1	(14.3)	1	(6.7)
**Renal failure**	Possibly related	-	-	-	-	1	(14.3)	1	(6.7)

* Abdominal pain possibly related to the procedure occurred in one patient. All other AEs occurred in one patient receiving FOLFIRINOX. In this patient, the AEs occurred 11 days after *siG12D-LODER^TM^* implantation or 5 days after initiation of FOLFIRINOX treatment and found by the DSMB to be unlikely related to the siRNA drug.

Six drug possibly-related AEs (Table 2) were reported by two patients (14.3%) in the 3mg treatment group. In one patient the two reported AEs (abdominal pain and constipation) were of grade 1 and were defined one day after *siG12D-LODER^TM^* implantation. Four remained drug possibly-related AEs were reported by one patient and occurred 11 days post *siG12D-LODER^TM^* implantation and four days after initiating FOLFIRINOX. Of these, one was grade 2 renal failure. The remaining three AEs (grade 4 pancytopenia and grade 3 abdominal pain and colonic obstruction), were considered as possibly related to the study procedure and possibly related to the FOLFIRINOX chemotherapy, while are unlikely to be related to the siRNA drug itself, as concluded by the Data Safety Monitoring Board (DSMB). Overall, siG12D was safe and well tolerated in doses of up to 3mg.

### Efficacy

**Figure 3:**
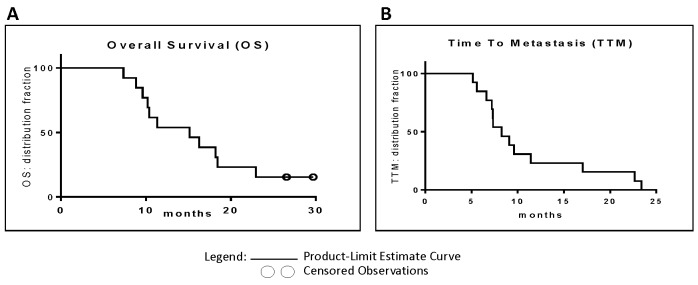
**A.** Overall survival: Kaplan-Meier curves depict OS of *siG12D-LODER^TM^*-treated patients. The two open circles mark patients who were still alive at the time of analysis. **B.** Time To Metastasis: Kaplan-Meier curves depict TTM of *siG12D-LODER^TM^*-treated patients.

Efficacy results in this study are based on single dosing only (without repeat dosing after four months). Median OS for all patients was 15.12 months with 95% confidence intervals (CI) of 10.19 to 18.44 months. One year, 18 months and two years survival were 53.8%, 38.5% and 15.4% respectively. Data are based on predictive Kaplan-Meier analysis, valid to December 2014, where two (from Cohort III) out of 13 patients were still alive (Figure 3A). The first patient to die was after 7.36 months. Significant differences of OS between the three dose groups were not observed. Of note, in FOLFIRINOX-treated group (*n* = 3), median OS was longer than 27 months (while submitting this manuscript, two patients are alive, more than 27 and 30 months).

None of the patients showed tumor progression (PFS) at all analyzed time points (Figure 5).

The TTM was over 5.16 months in all patients. Median TTM was 8.25 months with 95% CI of 7.20 to 11.40 months. At 18 months TTM was 15.4%. Data is based on predictive Kaplan-Meier analysis valid to December 2014, (Figure 3B). Significant differences of TTM between the three dose groups were not observed. In FOLFIRINOX-treated group, median TTM was 22.65 months.

**Figure 4:**
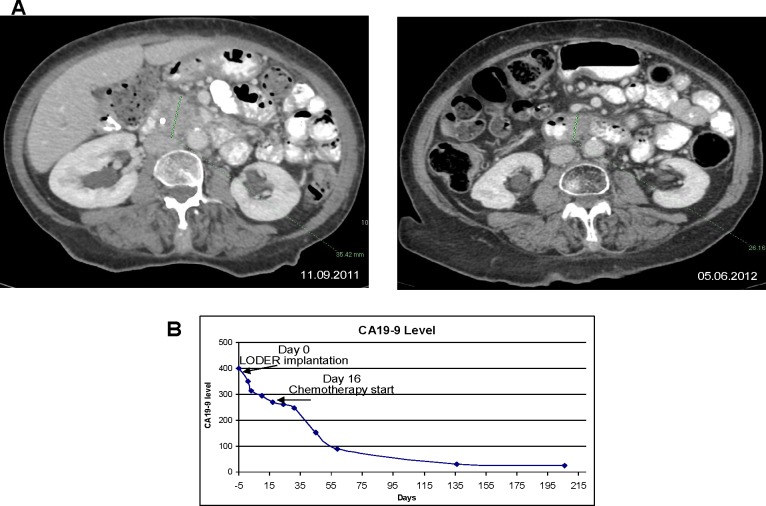
Anti-tumor effect of combination treatment with *siG12D-LODER^TM^* in locally advanced non-operable PAC in a patient **A.** left panel: a CT scan was performed prior to the implantation of *siG12D-LODER^TM^* using EUS; tumor measures 35.42mm in longest diameter; right panel: nine months later a significant tumor mass reduction is shown on a follow-up CT scan, tumor measures 26.16mm in the longest diameter. **B.** The level of CA19-9 in blood, showing 23% decrease immediately after *LODER^TM^* insertion, prior SOC treatment.

In particular, we present a case of one of the patients from the low dose group. The patient initiated Gemcitabine chemotherapy 16 days after the *siG12D-LODER^TM^* implantation. The treatment was well tolerated, with no significant side-effects. The serum-based tumor-marker CA19-9 decreased by 22.4% following the *siG12D-LODER^TM^* implantation before the administration of the first line chemotherapy treatment, and eventually reached normal values (Figure 4B). This patient then received local radiation treatment. A CT scan performed nine months post insertion of siG12D showed a significant decrease in tumor size (Figure 4A). This patient had 17.03 months TTM and died 18.21 months after enrollment to the study.

### CT analysis

**Figure 5:**
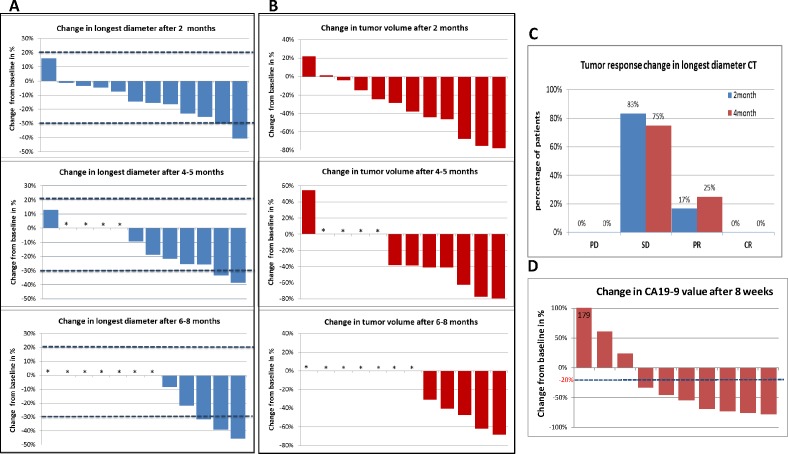
CT changes from base-line: The difference in the CT measurements shown as a waterfall plot **A.** Change in longest diameter (LD) 2, 4 and 6-8 months after *siG12D­-LODER^TM^* implantation. (*) marks the cases for which data after 4 months or later were not available. **B.** Change in tumor volume 2, 4 and 6-8 months after *siG12D-LODER^TM^* implantation. (*) marks the cases for which data after 4 months or later were not available. **C.** Percentage of patients who showed progression of disease (PD); stable disease (SD); partial response (PR) or complete response (CR), based on the changes in LD according to the RECIST 1.1 guidelines. **D.** CA19-9 changes after 8 weeks: The graph shows waterfall plot of changes from baseline in the levels of the CA19-9 tumor marker.

Figure 5A and 5B display CT response in the longest diameter (LD, based on direct measurements) and in volume (based on 3D reconstruction), respectively, of tumors in 12 patients at 2 months following the *siG12D-LODER^TM^* insertion and after approximately four months and 6-8.5 months in patients were data was available. Measurements of changes in LD show that none of the tumors progressed according to RECIST1.1 (above 20%); two patients showed decrease in LD below 30%. Analysis at four months and at 6-8.5 months after the procedure showed that two of eight (25%) and three of five (60%) patients achieved partial response, respectively. Two patients (13.3%) had stable disease (one each in the 0.75mg and the 3mg treatment groups). To note, none of the patient for whom CT data is available showed tumor progression (LD>20%) in the first 8 months (PFS = 100% at the first ∼6-8 months). Figure 5B shows a significant decrease in tumor volume in most patients.

### Post-therapy CA19-9 changes

At enrollment, abnormal elevated tumor marker CA19-9 levels (>37 U/mi) were found in 10 patients among the 13 patients who were analyzed for efficacy. Decrease of >20% is significant, following Ziske et al.'s report demonstrating that the decrease of CA19-9 >20% after 8 weeks of chemotherapy is able to separate patients into groups with significantly different survival times [21]. In this study, decrease in tumor marker CA19-9 was observed in 70% of patients (7/10). The data of 8-weeks follow-up showed significant decrease in all the 7 patients (Figure 5D).

## DISCUSSION

Pancreatic cancer is still one of the major challenges in clinical oncology. Mutant KRAS is a driving oncogene in the majority of human pancreatic cancer cases [14].

We have met this challenge by developing a therapeutic platform for local and prolonged delivery of siRNA. Our report describes an efficient, clinically applicable siRNA delivery method that overcomes the major obstacles of toxicity and organ accessibility. Notably, our approach enabled the conversion of KRAS from a non-druggable to a potentially druggable cancer target.

The present study evaluated single administration of three escalating doses of siG12D together with standard of care chemotherapy in patients with locally advanced inoperable pancreatic cancer. The primary endpoints were to evaluate the safety and toxicity of siG12D in combination with chemotherapy. No DLTs were observed during the study, therefore no MTD could be determined, and the highest dose administered and well tolerated was 3mg. Most AEs were grade 1 and 2, transient, and not related to the study drug or to the study procedure. In the 3mg treatment group, the AEs were related to either the implantation procedure, or the chemotherapy treatment (FOLFIRINOX).

The results of the Phase 1/2a study demonstrated a median OS of 15.12 months, a median TTM of 8.25 months; 18 months OS was 38.5% and TTM 15.4%. Most patients had stable disease and two patients had demonstrated partial responses. Six to 8.5 months after *LODER^TM^* insertion, 60% of the patients of whom CT data is available achieved partial response, and 40% had stable disease. No dose response was observed between the dose of siG12D and OS or TTM. Of note, two patients from the high dose group are still alive (27 and 30 months).

In this study we present the affectivity of LODER*^TM^* for RNAi based treatment for solid tumors. Our strategy combines selection of cancer-driver target with prolonged delivery to cancer cells. The LODER*^TM^* platform is based on encompassing of siRNA within a miniature biodegradable polymeric matrix that protects and enables the release of the siRNA drug for an extended duration of time regionally within tumor tissue. The LODER*^TM^* is specifically designed to be inserted with existing EUS biopsy procedures. We showed the efficacy of siG12D drug, and that the innovative *siG12D-LODER^TM^* delivery system is efficient, site-specific, and safe.

In summary, the concomitant administration of *siG12D-LODER^TM^* and chemotherapy was safe and well tolerated. The prolonged clinical benefit warrants further evaluation of this agent in combination with chemotherapy. A multinational randomized Phase 2b clinical trial is currently in progress (NCT01676259).

## PATIENTS, MATERIALS AND METHODS

The Phase 1/2a study (NCT01188785) was conducted at three medical centers in Israel, namely the Chaim Sheba Medical Center, Tel HaShomer, the Hebrew University-Hadassah Medical Center, Ein Kererm Jerusalem, and the Shaare Zedek Hospital, Jerusalem. The *siG12D-LODER*^TM^ polymeric implants were provided by Silenseed Ltd. Patients were enrolled between July, 2010 and May, 2012 in accordance with the principles of Good Clinical Practice and the Declaration of Helsinki. The protocol was approved by the Israeli Ministry of Health and Local Institutional Review Boards, and patients provided written informed consent before participation.

### Study population

Patients with unresectable, locally advanced confirmed or highly suspected adenocarcinoma of the pancreas, or resectable tumors in patients who defer surgery due to a high surgical risk (e.g. coagulopathy or severe congestive heart failure) were enrolled. No upper age limit was applied. Eligibility criteria included patients eligible to receive SOC as first line treatment in accordance with treating physician recommendation, target tumor that was accessible for intra-tumoral administration by EUS guidance as determined by the radiologist/gastroenterologist performing the EUS insertion, Karnofsky performance status of ≥ 70%, life expectancy of ≥ 3 months, serum creatinine < 2.0 mg/dL, PT - INR < 1.5, absolute neutrophil count (ANC) > 1,000 × 10^3^ cells/mL, platelets ≥ 75,000/mL, hemoglobin ≥ 10mg/dL, no other malignancy present that would interfere with the current intervention and have measurable disease. Patients were excluded if they had distant metastases, including liver, lung, or lymph nodes, peritoneal spread or malignant ascites. Other exclusion criteria included clinically significant pancreatitis within 12 weeks of treatment, medical condition that would preclude both percutaneous and endoscopic guided delivery of the any concurrent medical illness or medical condition that would compromise patient safety or the objectives of the study as determined by a study investigator, or history of bleeding coagulopathy.

### Study design

This study was an open label, escalating single dose Phase 1/2a multi-center trial. Men and women ≥18 years old diagnosed with non-operable PDAC or with high evidence of PDAC with no evidence of distant metastasis were screened for eligibility. Metastases presence was assessed by CT scan. Adenocarcinoma of pancreas was confirmed by biopsy.

In this study, single dosing (single time) of escalating doses of *siG12D-LODER^TM^* (0.025, 0.75 and 3mg) were administered by a standard procedure EUS guidance. *siG12D-LODERs* were inserted into the tumor using the FDA approved equipment, namely EchoTip^®^ Ultra (Cook Medical) or the equivalent Expect 19 Flex Biopsy needle (Boston Scientific) (Figure 1). After insertion patients were monitored carefully for signs of systemic or local treatment-related toxicity. Scheduled physical and clinical examinations, vital signs (estimated by Karnofsky performance status (PS)), laboratory tests, tumor marker CA19-9, abdominal CT and disease assessments were performed during the course of the trial. Following the *siG12D-LODER^TM^* insertion Gemcitabine 1000mg/m^2^ IV was given on a weekly basis until disease progression. One patient received Gemcitabine + Erlotinib + Oxaliplatin (1000mg/m^2^, 100mg and 100mg/m^2^ IV respectively) for 3 weeks, followed by Gemcitabine alone, 1000mg/m^2^ IV on a weekly basis. The RP2D (recommended Phase 2b dose) was further examined in highest dose cohort in combination with modified FOLFIRINOX (Oxaliplatin 85mg/m^2^, Irinotecan 150mg/m^2^ followed by a Fluorouracil continuous IV infusion of 2,400mg/m^2^ 46 hours every two weeks). Active follow-up period was eight weeks after placement of *siG12D-LODER^TM^* and long-term survival follow-up was performed until death. After two months, CT scan was performed as a SOC at each study site; the results were presented in a blinded to the sponsor for analysis.

### Safety assessments

Toxicity was evaluated according to the NCI Common Terminology Criteria for Adverse Events (CTCAE), Version 4.0. A minimum of four patients were entered at each dose level. All four subjects were followed until they completed the cycle of therapy and subsequent enrolment of new cohorts was based on the toxicity assessment in that first cycle and the documentation of any dose limiting toxicities. A two week gap was enforced between cohorts. Before opening the next higher dose level, all toxic effects at the preceding dose level were reviewed and expansion or escalation was undertaken as appropriate. Meetings between investigators were organized as required.

### Efficacy measurements

Antitumor activity was assessed based on changes in CA19-9 and imaging. Changes were based on RECIST 1.1 criteria. Imaging was performed at baseline and at 12 weeks, and in most patients subsequently after 4 months and 6 months. Progression free survival (PFS) was defined based on the CT data (based on RECIST1.1) as the first time to show longest diameter (LD) > 20%. Overall survival (OS) was defined from the date of diagnosis until death from any cause. TTM was defined from the date of diagnosis until detection of metastasis. Data is based on predictive Kaplan Meier analysis valid until December 2014, when two (from Cohort III) out of 13 patients were still alive, and one having not demonstrated disease progression (after 27 months).

### Statistical analysis

All measured variables and derived parameters were listed individually and, if appropriate, tabulated by descriptive statistics. Descriptive statistics and summary tables were provided giving sample size (n), absolute and relative frequency of categorical variables and sample size, arithmetic mean, standard deviation, coefficient of variation (if appropriate), median, minimum and maximum percentiles and 95% confidence interval (CI) for means of continuous variables. All two-tailed statistical tests with a *P* ≤ 0.05 were considered significant. All adverse events were classified by System Organ Class and preferred terminology according to MedDRA dictionary and were summarized in cross tables by severity and dose group. All tests applied were two-tailed, and *P* values ≤ 0.05 were considered significant. The data were analyzed using the SAS^®^ software version 9.1 (SAS Institute, Cary, North Carolina).

All authors had access to the study data, and reviewed and approved the final manuscript.

## SUPPLEMENTARY MATERIAL FIGURES


