# Supplementary material for: RNAi therapy targeting KRAS in combination with chemotherapy for locally advanced pancreatic cancer patients
Source: Oncotarget. 2015 May 19;6(27):24560–70. doi: 10.18632/oncotarget.4183 (PMC4695206; doi:10.18632/oncotarget.4183)
Supplement: Supplementary file 1 [file oncotarget-06-24560-s001.pdf]

RNAi therapy targeting KRAS in combination with chemotherapy for locally advanced pancreatic cancer patients

Supplementary Material

A

human dose	mouse dose
mg	µg
**3mg**	**12ug**
1.25	5
0.125	0.5
0.025	0.1
0.0025	0.01

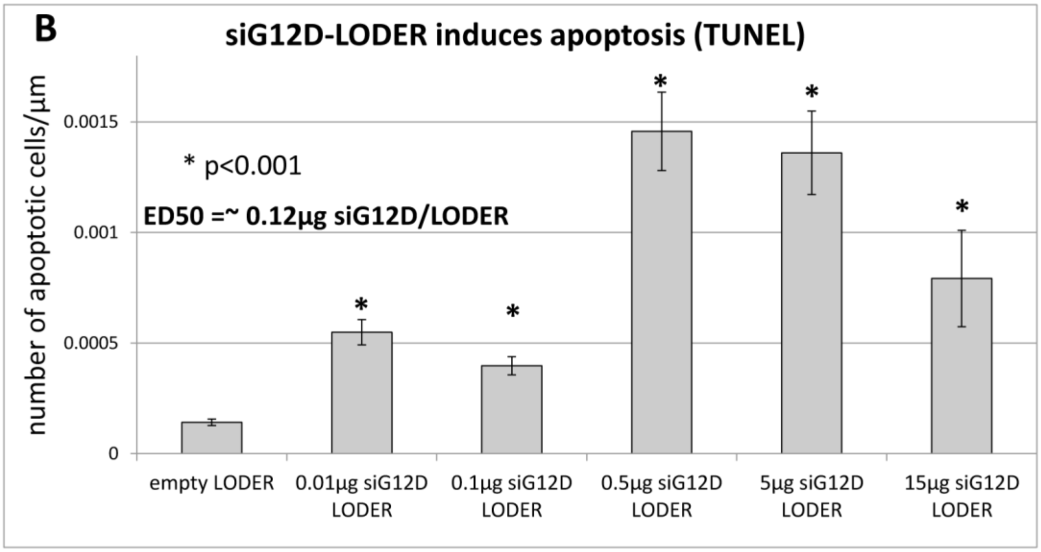

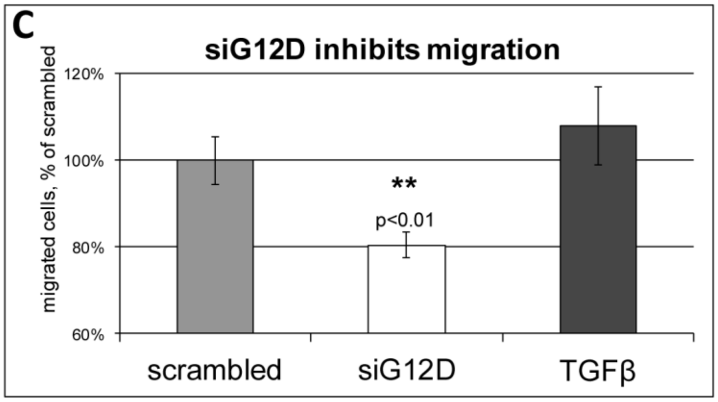

D

Fraction of mice having metastases in liver, lung of spleen	
u/t	50%
empty LODER	63%
**siG12D LODER**	**0%**

Supplementary Figure 1. (A) The table summarizes the equivalent human-mouse siG12D doses. **(B)** Subcutaneous tumors of pancreatic PancO2 origin were treated with empty *LODER*^{TM} or *LODER*^{TM} containing siG12D at noted doses. Four days post implantation, mice were sacrificed, tumor tissue was formalin-fixed, paraffin embedded and cut to a slices of 5 µm and stained to detect apoptotic tumor cells (TUNEL). Average number of apoptotic tumor cells per micrometer was calculated using NIS-element computer software. The graph shows the average number of apoptotic tumor cells per micrometer. The "median effective dose" (ED50) was calculated based on linear regression. **(C) siG12D inhibits migration of pancreatic tumor cells.** Pancreatic tumor cell migration was assessed *in vitro* using a transwell-chamber. Cells were transfected with siG12D or with scrambled, non-targeting siRNA (scrambled). TGFβ was used as EMT-positive control. The graph represents the percentage of migrated cells. **(D) siG12D inhibits formation of metastases**. Orthotopic pancreatic tumors were treated with empty LODERs, siG12D-LODERs or left untreated. Macrometastasis formation was assessed one month after LODER implantation. The table summarizes the percentage of mice which developed metastases in liver, lung and spleen.

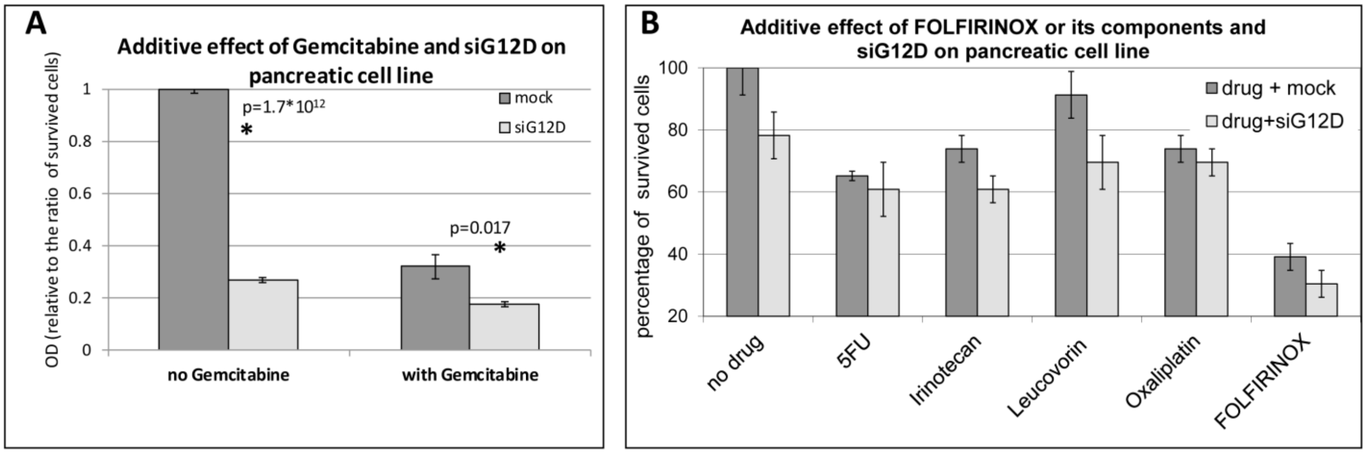

Supplementary figure 2. Additive effect of siG12D and chemotherapy drugs. Additive effect of siG12D and SOC was assessed in vitro by cell (human pancreatic cancer cell line Panc1) viability assay. Here cells were transfected with siG12D or mock-transfected. The effect was assessed with or without SOC: Gemcitabine (A) of the components of FOLFIRINOX (B).
